# Neuroanatomical patterns of dementia risk in autism spectrum disorder

**DOI:** 10.3389/fnagi.2026.1771822

**Published:** 2026-04-02

**Authors:** Young Seon Shin, Jingying Wang, Stormi L. Pulver, Ann-Marie Orlando, Regilda A. Romero, Carolina R. Cuomo, Isabella Valiente Lauzan, Tyler Dentry, Desirae J. Shirley, Danielle Christensen, Kathryn E. Unruh, Cassandra J. Stevens, Walker S. McKinney, Matthew W. Mosconi, David E. Vaillancourt, Zheng Wang, Stephen A. Coombes

**Affiliations:** 1 Laboratory for Rehabilitation Neuroscience, Department of Applied Physiology and Kinesiology, University of Florida, Gainesville, FL, United States; 2 Neurocognitive and Behavioral Development Laboratory, Department of Applied Physiology and Kinesiology, University of Florida, Gainesville, FL, United States; 3 Department of Pediatrics, Emory School of Medicine, Atlanta, GA, United States; 4 Center for Autism and Related Disabilities (CARD), University of Florida, Gainesville, FL, United States; 5 UF Health Center for Autism and Neurodevelopment (UF Health CAN), University of Florida, Gainesville, FL, United States; 6 Department of Psychiatry, University of Florida, Gainesville, FL, United States; 7 Life Span Institute, Dole Human Development Center, University of Kansas, Lawrence, KS, United States; 8 Kansas Center for Autism Research and Training (K-CART), University of Kansas, Lawrence, KS, United States; 9 Department of Behavioral Medicine and Clinical Psychology, Cincinnati Children’s Hospital Medical Center, Cincinnati, OH, United States; 10 Life Span Institute, Dole Human Development Center, University of Kansas, Lawrence, KS, United States; 11 Department of Neurology and McKnight Brain Institute, University of Florida, Gainesville, FL, United States; 12 Department of Biomedical Engineering, University of Florida, Gainesville, FL, United States

**Keywords:** aging, autism spectrum disorder, cortical thickness, dementia, structure MRI

## Abstract

Autism spectrum disorder (ASD) is a neurodevelopmental disorder. While previous studies have reported a high prevalence of dementia diagnoses in the ASD population, the risk of dementia-related neurodegeneration remains poorly understood. This study aimed to assess dementia-sensitive composite measures of brain structure and brain age across the lifespan in an ASD cohort (ages 7–73) to investigate neuroanatomical features linked to neurodegenerative vulnerability. The composite score and brain age were not significantly different between diagnostic groups when analyses considered the entire cohort, nor when the cohort was split into subgroups based on age. However, age-effects were found for both groups, with negative relationships revealing a decrease in cortical thickness with increasing age. Subgroup analyses showed that the same negative relationship was evident in the old NT group but was absent in the old ASD group, even when controlling for clinical factors. Our findings are consistent with the safeguard or parallel development hypothesis and suggest that conventional dementia-like structural vulnerability is not widespread and generalizable in ASD. Our observations highlight the limitations of current dementia-sensitive composites in assessing neurodegenerative risk in ASD. Developing dementia-sensitive biomarkers for autistic individuals is critical to enhance diagnostic precision and identify clinically significant biomarkers in this cohort.

## 1 Introduction

Autism spectrum disorder (ASD) is a complex developmental condition that encompasses a broad range of challenges in communication, social interaction, and restricted and repetitive behaviors (American Psychiatric Association, 2013). Aging processes in autistic individuals have received limited research attention, particularly concerning the co-occurrence of age-related conditions such as dementia (Wang et al., 2024). Epidemiological studies have shown that autistic individuals are at higher risk for dementia (Croen et al., 2015; Hand et al., 2020; Vivanti et al., 2021). For instance, Vivanti et al. (2021) reported that early-onset dementia is twice as prevalent in autistic adults aged 30–64 years compared to the general population, with the average onset occurring approximately 5 years earlier in autistic individuals without intellectual disabilities (mean age of onset: 49.35 years in ASD vs. 53.77 years in the general population). The risk is even higher in autistic adults with intellectual disabilities, who exhibit a 2.6-fold greater prevalence and an earlier average onset of 47.51 years.

Overlapping behavioral and biological markers between ASD and dementia also have been identified (Croen et al., 2015; Ray et al., 2011; Rhodus et al., 2022; Rossignol and Frye, 2014; Sokol et al., 2006; Vivanti et al., 2021). Conditions such as anxiety, depression, and deficits in executive functions—including planning, flexibility and working memory—are common in dementia and are also frequently observed in autistic adults (Alzheimer’s Association, 2016; Braden et al., 2017). Additionally, elevated beta-amyloid precursor protein levels (Ray et al., 2011; Sokol et al., 2006) and the alleviation of these mental health and communication challenges with medications approved for Alzheimer’s disease (AD; Rossignol and Frye, 2014) in autistic individuals suggest potential shared pathways between ASD and dementia (Ghaleiha et al., 2013; Hertzman, 2003). These parallels raise critical questions regarding whether ASD and dementia share a neurobiological foundation or, conversely, whether ASD may exhibit a distinct neurodegenerative profile.

Pathologically, AD, the most common form of dementia, is characterized by the formation of amyloid-*β* plaques and neurofibrillary tangles, leading to neuronal damage, loss of brain tissue, and eventual death of nerve cells (Alzheimer’s Association, 2016; Braak et al., 2006; Mathis et al., 2003; Thal et al., 2002). The pattern of neurodegeneration typically begins in the medial temporal lobe, including the hippocampus, and spreads to other regions as the disease progresses (Braak et al., 2006; Tremblay et al., 2025). Biomarkers derived from neuroimaging, such as magnetic resonance imaging (MRI)-based measures of brain atrophy, have been developed to detect early pathological changes. MRI-based analyses of “AD signature” regions have shown promise in predicting disease stage and progression to mild cognitive impairment (MCI) or AD (Bottino et al., 2002; Dickerson et al., 2009; Liu et al., 2010; McEvoy et al., 2009; Schwarz et al., 2016). Recently developed composite scores of cortical thickness in AD-vulnerable regions, such as the entorhinal and temporal cortices, show correlations with disease severity and risk of progression from MCI to AD (Schwarz et al., 2016). The Schwarz composite (Schwarz et al., 2016), which is derived from cortical thickness, has demonstrated a strong correlation with AD pathology. Although this composite was developed in older non-autistic cohorts, whether they differ in autistic individuals given the higher prevalence rates of dementia in ASD remains an open question.

Multiple neurodevelopmental differences in autistic individuals have been documented, including patterns of early brain overgrowth, followed by a plateau, and subsequent decline during adolescence and young adulthood (Hazlett et al., 2011; Lange et al., 2015; Zielinski et al., 2014). These structural abnormalities suggest potential age-related changes in regions implicated in AD and related dementias. ASD-related brain atrophy such as cortical thinning has been observed in regions overlapping with those sensitive to AD pathology, such as temporal and parietal cortices in middle aged autistic adults (Braden et al., 2017; Wallace et al., 2010). This raises the possibility of shared or interacting mechanisms between neurodevelopmental disruptions in ASD and neurodegenerative processes underlying dementia. Investigating whether ASD-associated structural changes extend to regions associated with dementia may elucidate the intersection of these conditions, and potentially advance biomarker development and understanding of mechanisms associated with aging-related changes in ASD.

In the current study, we examined brain atrophy using a well-established dementia-sensitive composite measure (Schwarz et al., 2016) which quantifies cortical thickness in regions vulnerable to AD. We also calculated brain age. We first assessed the vulnerability to AD and brain age across the entire cohort, and then conducted *a priori* subgroup analyses by age. Considering accelerated decline in cognitive domains can begin in one’s 40s (Antal et al., 2025; Dohm-Hansen et al., 2024), participants were separated into three subgroups (old: > 40 years, middle: 21–40 years, and young: <= 20 years). This approach provides a comprehensive view of age-related neuroanatomical differences in ASD and their potential relevance to AD vulnerability.

## 2 Methods

### 2.1 Participants

Participant recruitment, screening and testing were conducted at the University of Florida (UF), the University of Kansas (KU), and the University of Texas Southwestern Medical Center (UTSW). All study protocols were approved by the institutional review boards at UF (201801378), KU (STUDY00140269), and UTSW and Children’s Hospital of Dallas (STU052013-4). Written informed consent was obtained from participants 18 years of age or older. For minors and adults under legal guardianship, consent was obtained alongside written consent from caregivers or conservators in accordance with the Declaration of Helsinki.

We recruited a total of 258 participants across three research sites. After excluding 17 participants due to poor MR image quality, the final cohort consisted of 241 participants, including 122 autistic individuals and 119 neurotypical (NT) controls. (Table 1). Autistic individuals were recruited through clinical programs, institutional research registries, community advertisements, and SPARK Research Match.^1^ NT participants were recruited via study flyers and word of mouth. The study included three sites (Supplementary Table 1): the University of Florida (UF) cohort (45 autistic and 60 NT participants), the University of Kansas (KU) cohort (58 autistic and 40 NT participants), and the University of Texas Southwestern (UTSW) cohort (19 autistic and 19 NT participants).

**Table 1:** Demographic and clinical characteristics in autistic individuals (ASD) and neurotypical controls (NT).

All participants				
	ASD mean (SD)	NT mean (SD)	t/χ^2^	*p*
N	122	119		
Age (years) Range	28.35 (16.15) 10–73	33.33 (17.31) 7–70	2.31	**0.022**
Sex (M/F)^a^	83/39	62/57	5.73	**0.017**
% of female	32.0%	47.9%		
Full-scale IQ	105.4 (15.45)	110.6 (12.77)	2.84	**0.005**
Verbal IQ^b^	104.1 (16.35)	108.6 (11.80)	1.62	**0.022**
Performance IQ^b^	105.2 (14.80)	109.6 (13.46)	1.64	**0.023**
ADOS-2 (CSS)^c^	6.78 (3.36)	-		-
ADOS-2 (total raw)^d^	10.81 (3.29)	-		-
Total intracranial volume (cm^3^)	1,573 (165.1)	1,523 (161.1)	2.39	**0.018**
Old group (Age > 40)				
	ASD mean (SD)	NT mean (SD)	t/χ^2^	*p*
N	29	41		
Age (years) Range	52.72 (8.15) 41–73	53.95 (7.95) 41–70	0.63	0.533
Sex (M/F)^a^	17/12	17/24	1.37	0.241
% of female	41.4%	58.5%		
Full-scale IQ	108.0 (12.68)	107.5 (11.69)	0.16	0.877
Verbal IQ	107.6 (12.17)	105.8 (9.73)	0.67	0.505
Performance IQ	106.7 (14.07)	107.7 (13.59)	0.29	0.775
ADOS-2 (total raw)^e^	9.10 (3.16)	-		-
SRS self T-score^f^	77.78 (7.53)	45.77 (4.83)	19.25	**< 0.001**
RBS-R^g^	41.82 (29.00)	2.66 (3.03)	7.12	**< 0.001**
Total intracranial volume (cm^3^)	1,522 (186.1)	1,478 (157.0)	1.05	0.298
Middle group (20 < Age ≤40)				
	ASD mean (SD)	NT mean (SD)	t/χ^2^	*p*
N	36	43		
Age (years) Range	30.31 (5.99) 21–40	29.33 (5.493) 21–40	0.75	0.454
Sex (M/F)^a^	25/11	26/17	0.35	0.552
% of female	30.6%	39.5%		
Full-scale IQ	106.2 (18.49)	112.9 (12.45)	1.85	0.069
Verbal IQ^h^	106.3 (18.04)	111.4 (13.09)	1.39	0.169
Performance IQ^h^	103.1 (15.54)	111.7 (13.74)	2.52	**0.014**
ADOS-2 (CSS)^i^	14.33 (3.21)	-		-
ADOS-2 (total raw)^j^	11.43 (2.44)	-		-
Total intracranial volume (cm^3^)	1,612 (149.2)	1,554 (159.9)	1.66	0.102
Young Group (Age ≤20)				
	ASD mean (SD)	NT mean (SD)	t/χ^2^	*p*
N	57	35		
Age (years) Range	14.72 (2.97) 10–20	14.09 (3.82) 7–20	0.84	0.406
Sex (M/F)^a^	41/16	19/16	2.25	0.134
% of female	28.1%	45.7%		
Full-scale IQ	103.6 (14.65)	111.3 (13.98)	2.53	**0.014**
Verbal IQ^k^	100.3 (16.89)	108.6 (11.93)	2.42	**0.018**
Performance IQ^k^	105.8 (14.84)	109.2 (12.81)	1.02	0.312
ADOS-2 (CSS)^l^	6.09 (2.43)	-		-
Total intracranial volume (cm^3^)	1,574 (159.4)	1,536 (160.3)	1.09	0.281

a Chi-square (χ^2^) statistics.

b included 108 in ASD and 109 in NT.

c ADOS-2 CSS score was used for those age less than 31 including 3 participants in the UF cohort, 30 participants in the KU cohort, and 3 participants in the UTSW cohort.

d ADOS-2 total raw score was used for those ages over 30 including 42 participants from UF cohorts.

e ADOS-2 total raw score was used for those ages over 30 including 28 participants from UF cohorts.

f included 27 ASD and 35 NT from UF cohorts.

g included 28 ASD and 41 NT from UF cohorts.

h included 34 ASD and 42 NT.

i ADOS-2 CSS score was used for whose age less than 31 including 3 participants from the UTSW cohort.

j ADOS-2 total raw score was used for those ages over 30 including 14 participants from the UF cohort.

k included 45 ASD and 26 NT.

l ADOS-2 CSS score was used for those ages less than 31 including 3 participants in the UTSW cohort and 30 participants in the KU cohort.*p*-values in bold indicates statistical significance (*p* < 0.05).

Autism diagnosis was based on the criteria in the Diagnostic and Statistical Manual of Mental Disorders, 5th Edition (DSM-5; American Psychiatric Association, 2013) for UF and KU cohorts, and the IV Edition (DSM-IV; American Psychiatric Association, 1995) for the UTSW cohort. In the UF cohort, autistic participants were screened using the Autism Spectrum Quotient for Adults (AQ; Baron-Cohen et al., 2001) and the Social Responsiveness Scale Adult Self-Report (SRS-2; Constantino and Gruber, 2012). Individuals with scores ≥ 32 on the AQ and ≥ 65 on the SRS-2 were further evaluated using the Autism Diagnostic Observation Schedule, Second Edition (ADOS-2; Lord et al., 2012). The diagnosis of ASD was confirmed based on ADOS-2 and expert clinical opinion using DSM-5 criteria (American Psychiatric Association, 2013). Additionally, 10 autistic participants did not meet the criteria for either AQ or SRS-2 but they were confirmed to have a clinical diagnosis of ASD based on ADOS-2 scores > 7 and research clinician evaluations (AMO and RAR). For the KU and UTSW cohorts, the Social Communication Questionnaire (SCQ; Rutter et al., 2003) was used for screening, and autistic individuals with SCQ scores > 15 were recruited. The ASD diagnosis was confirmed using the ADOS-2 (Lord et al., 2012), the Autism Diagnostic Interview-Revised (ADI-R; Rutter, 2003), and expert clinical evaluation based on DSM IV (UTSW; American Psychiatric Association, 1995) or DSM-5 criteria (KU; American Psychiatric Association, 2013). Autistic individuals were excluded if they had any known genetic or metabolic disorder associated with ASD (e.g., fragile X syndrome, Phelan-McDermid syndrome, tuberous sclerosis, or Burnside-Butler syndrome).

For the UF cohort, prospective NT participants were recruited if they scored ≤ 24 on the AQ and < 60 on the SRS-2. In the KU and UTSW cohorts, NT participants with scores < 8 on the SCQ were invited to the study. Exclusion criteria for NT participants included: (1) recent history of or current psychiatric conditions (e.g., schizophrenia, bipolar affective disorder, obsessive-compulsive disorder); (2) a family history of psychiatric disorder in first-degree relatives; (3) a family history of ASD or other neurodevelopmental disorder in first- or second-degree relatives. All participants had corrected or uncorrected visual acuity of at least 20/40.

### 2.2 MRI data acquisition

For the UF cohort, anatomic images were obtained with a 3 T Siemens Prisma with a 64-channel head coil. A T1-weighted magnetization prepared rapid gradient echo (MPRAGE) sequence was used for 49 participants with the following parameters: repetition time (TR) = 2000 ms, echo time (TE) = 2.99 ms, flip angle = 8 degrees, field of view (FOV) = 256 × 256, matrix = 320 × 320, 208 sagittal slices, voxel size = 0.8 × 0.8 × 0.8 mm^3^. A T1-weighted accelerated sagittal MPRAGE sequence was used for 56 participants with the following parameters: TR = 2,300 ms, TE = 2.98 ms, flip angle = 9°. FOV = 253 × 270, matrix = 256 × 256, 176 sagittal slices, voxel size = 1.0 × 1.0 × 1.0 mm^3^. For the KU cohort, anatomic images were acquired with a 3 T Simens Skyra and a 32-channel head coil. The T1-weighted MPRAGE sequence was collected with the following parameters: TR = 2,300 ms, TE = 2.95 ms, flip angle = 9°. FOV = 253 × 270, matrix = 240 × 256, 176 sagittal slices, voxel size = 1.05 × 1.05 × 1.2 mm^3^. For the UTSW cohort, anatomic images were obtained with a 3 T Phillips Achieva with a 32-channel head coil. The T1-weighted MPRAGE sequence was collected with the following parameters: TR = 8.1 ms, TE = 3.73 ms, flip angle = 12°, FOV = 256 × 204 mm, matrix = 256 × 204, 160 sagittal slices, voxel size = 1.0 × 1.0 × 1.0 mm^3^.

### 2.3 Imaging processing

T1-weighted images were processed for volumetric segmentation and cortical reconstruction using FreeSurfer 7.2.0 (Fischl, 2012)^2^ on a Linux-based computing system. The image processing followed the default recon-all pipeline, which includes steps such as motion correction, intensity normalization, transformation to Talairach space, skull stripping, brain extraction, white matter segmentation, and cortical parcellation. Prior to further analysis, all processed images underwent rigorous quality assurance through slice-by-slice visual inspection. Manual corrections were performed using the Recon Edit tool in FreeSurfer where necessary. A total of 11 researchers participated in the review process, evaluating images across axial, coronal, and sagittal planes. Images with significant segmentation inaccuracies were corrected and subsequently cross-checked by a separate researcher to ensure consistency. Cases with severe errors were excluded from the study. A total of 17 (7.39%) participants were excluded from the 258 eligible cases. The current study reports results from 241 participants.

To harmonize imaging data from the four sequences, ComBat harmonization^3^ was employed. The ComBat tool effectively mitigates nuisance variability associated with scanners, while preserving biologically meaningful variability (Fortin et al., 2018), ensuring consistency across datasets from different imaging platforms.

Cortical thickness was calculated in AD sensitive areas including the entorhinal cortex, middle temporal gyrus, inferior temporal gyrus, inferior parietal cortex, fusiform gyrus, and precuneus (Schwarz et al., 2016). These measures were used to calculate the Schwarz composite, which was designed to distinguish individuals with MCI and AD from clinically healthy individuals (Schwarz et al., 2016). The Schwarz composite was calculated by the following Equation 1:


$$C_{\text{Schwarz}}=\frac{\sum_{k=1}^{n}t_{k}}{n}$$ (1)

where $C_{\text{Schwarz}}$ represents the Schwarz composite score, n represents the number of ROIs included in the Schwarz composite (bilateral entorhinal cortex, middle temporal gyrus, inferior temporal gyrus, inferior parietal cortex, fusiform gyrus, and precuneus), and $t_{k}$ represents the cortical thickness of each ROI.

### 2.4 Brain-age

We calculated brain-age, to estimate and compare the brain’s biological age to an individuals’ chronological age, to further investigate brain structure in ASD (Biondo et al., 2022; Gaser et al., 2013). Brain-age was estimated using BrainAgeR version 2.1^4^ (Cole et al., 2018) which was trained on 3,377 healthy individuals (age = 40.6 ± 21.4, 18–92 years) and tested on 857 healthy individuals (age = 40.1 ± 21.8, 18–90 years). This metric is well supported showing strong associations with chronological age and predicted brain age in adults (*r* = 0.973). BrainageR generated a brain age estimate from raw T1-weighted images using SPM12 (University College London, London, United Kingdom)^5^ to segment and normalize probability maps for gray matter, white matter, and cerebrospinal fluid (CSF). Principal component analysis was then implemented, and 435 principal components were retained to predict biological age with kernlab^6^ ((Karatzoglou et al., 2004).

### 2.5 Statistical analyses

Statistical analyses were conducted using R version 4.5.1 (R Core Team, 2025). We set the statistical significance to *p* < 0.05.

#### 2.5.1 Demographic and clinical information

Group differences in demographic and clinical information were assessed using independent samples t-tests. These analyses were conducted within the entire cohort and then separately within each age-group (old: > 40 years, middle: 21–40 years, and young: <= 20 years).

#### 2.5.2 Schwarz composite

##### 2.5.2.1 Whole cohort analyses

We conducted a permutation two-sample t-test with 5,000 permutations to compare Schwarz composite scores between ASD and NT groups. Permutation multiple regression analyses with 5,000 permutations were conducted to examine age effects within each diagnostic group. We further investigated these effects within each ROI included in the Schwarz composite. For ROI-level analyses, multiple comparisons were corrected by implementing the false discovery rate (FDR; Benjamini and Hochberg, 1995). All whole-cohort analyses were repeated controlling for FSIQ.

##### 2.5.2.2 Age-group analyses

Given that the Schwartz composite was developed for adults, we conducted permutation two-sample t-tests and permutation multiple regression within each age-group (old, middle, young). We conducted further regression analyses in the old age-group, including: (1) For each ROI, applying FDR correction (Benjamini and Hochberg, 1995); (2) examining Schwarz composite and age in the ASD group while controlling for clinical scores (SRS-2 and RBS-R), and; (3) ROI-based analyses with FSIQ included as a covariate with FDR correction.

#### 2.5.3 Brain age

##### 2.5.3.1 Whole cohort analyses

ANCOVA with 5,000 permutations was conducted to test group differences, with predicted age difference (ΔAge, predicted brain age—chronological age) as the dependent variable and group as the independent variable. Chronological age was included as a covariate, following recommendations from BrainAgeR (See Footnote 4). Additionally, ANCOVA with 5,000 permutation was conducted with ΔAge, while chronological age and FSIQ were included as covariates.

##### 2.5.3.2 Age-group analyses

Given that the brain age algorithm was developed for adults, permutation ANCOVAs (5,000 permutation) were conducted within each age-group to test for group differences in ΔAge with chronological age as a covariate. Finally, we repeated these analyses including FSIQ as an additional covariate.

## 3 Results

### 3.1 Demographic and clinical information

Demographic and clinical data are summarized in Table 1 for the whole cohort (top panel), and separately for each age group (bottom 3 panels). Data disaggregated by site is shown in Supplementary Table 1. For the full cohort, age, sex, full-scale IQ (FSIQ), verbal IQ (vIQ), performance IQ (pIQ) and total intracranial volume (TIV) were significantly different between ASD and NT groups (all *ps* < 0.05). ASD individuals had lower FSIQ (*p* = 0.005), vIQ (*p* = 0.022), and pIQ (*p* = 0.023) and higher TIV (*p* = 0.018) compared to the NT group.

The old age-group included 29 ASD and 41 NT participants. There were no significant differences between ASD and NT groups in age, sex, FSIQ, vIQ, pIQ, and TIV in the old age-group. ASD participants had higher SRS self-report T-scores and RBS-R scores (all *p*s < 0.001). The middle age group included 36 ASD and 43 NT participants. ASD participants had lower pIQ compared to NT participants (*p* = 0.014). All other comparisons were not significant. Finally, the young age-group included 57 ASD and 35 NT participants. ASD participants in the young age-group had lower FSIQ and vIQ (*p* = 0.014 and 0.018, respectively) compared to NT participants. All other comparisons were not significant.

### 3.2 Schwarz composite

**Figure 1:**
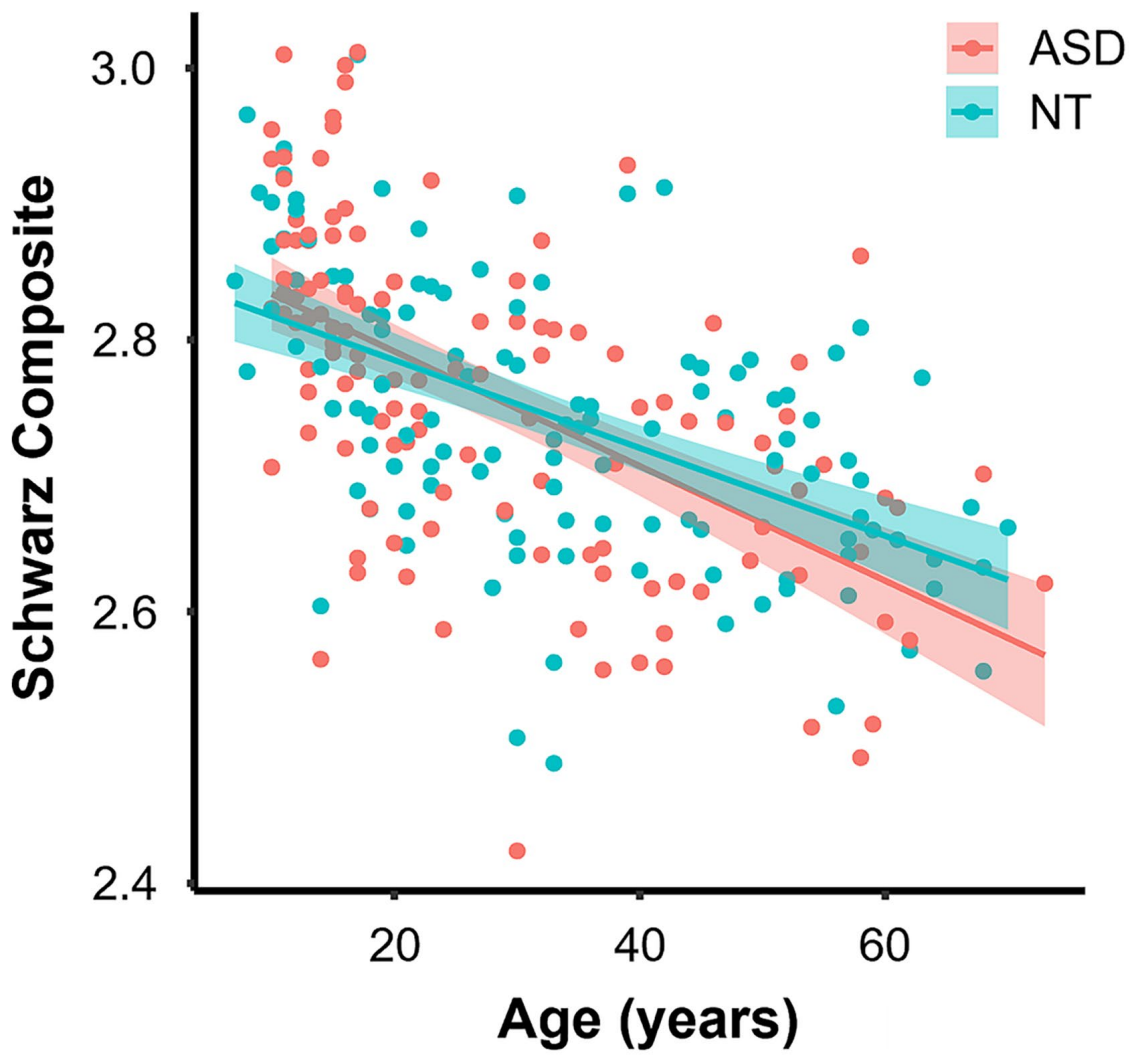
Scatter plot showing the relationship between age and Schwarz composite score (red—ASD, sky-blue—NT). Solid lines represent the linear regression fit for each group with shaded 95% confidence interval.

Across the whole cohort, the Schwarz composite did not significantly differ between ASD and NT groups, t(234.98) = 0.993, *p* = 0.322. However, a regression analysis of the Schwarz composite on age revealed a significant negative relationship (Figure 1; β = − 0.005, *t* = −5.73, *p* < 0.001), indicating that Schwarz composite scores declined with increasing age. Neither the main effect of diagnostic group ( β = − 0.013, *t* = −1.04, *p* = 0.302) nor the diagnostic group by age interaction ( β = − 0.0005, *t* = −1.37, *p* = 0.174) was significant. When separate regressions were conducted within each diagnostic group, both the ASD group ( β = − 0.004, *t* = −7.57, *p* < 0.001) and the NT group ( β = − 0.003, *t* = −7.06, *p* < 0.001) exhibited a significant age effect.

Further regression analyses were conducted separately within each diagnostic group to examine associations between age and the ROIs that are included in the Schwarz composite. In the ASD group, cortical thickness in 10 regions was negatively associated with age (bilateral middle temporal gyrus, inferior temporal gyrus, inferior parietal cortex, fusiform gyrus, and precuneus), with all these regions surviving FDR correction (*p*_FDR_s < 0.001). No age effect was evident in bilateral entorhinal cortex (both p_FDR_s = 0.159). In the NT group, cortical thickness was negatively associated with age in all regions: left entorhinal cortex (*p*_FDR_ = 0.001), right entorhinal cortex (*p*_FDR_ = 0.002), and bilateral middle temporal gyrus, inferior temporal gyrus, inferior parietal cortex, fusiform gyrus, and precuneus (all *p*_FDR_s < 0.001). Detailed regression statistics are provided in Supplementary Table 2.

**Figure 2:**
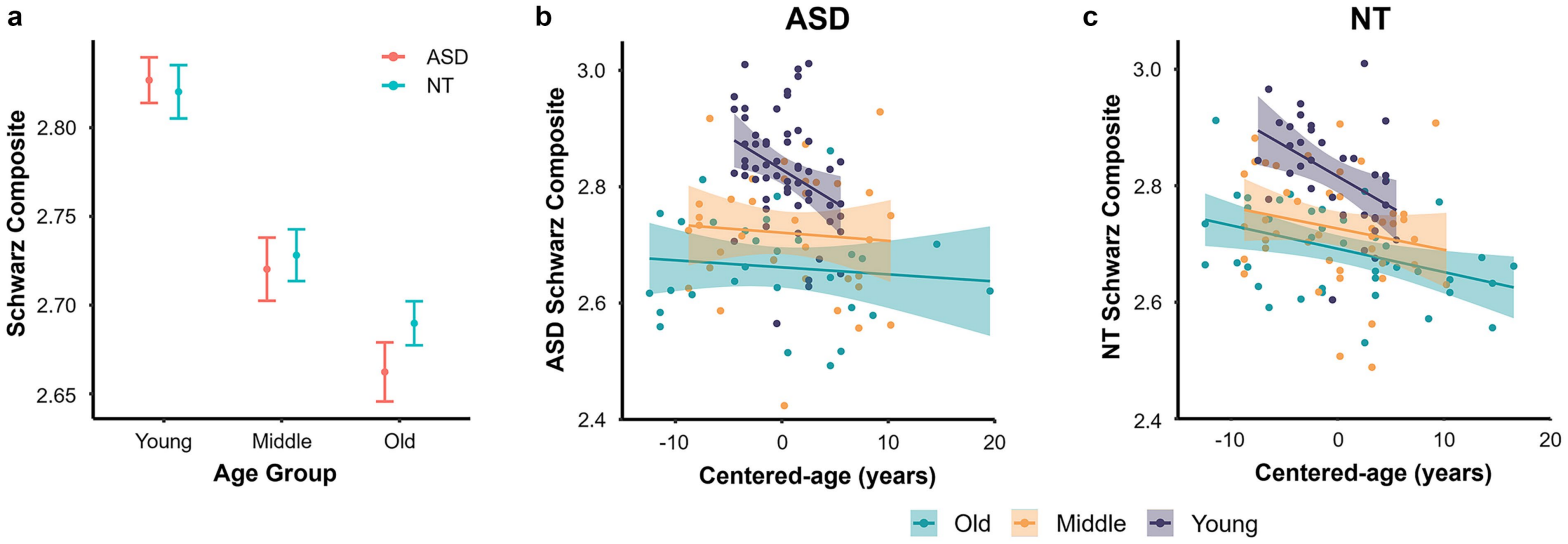
**(a)** Mean Schwarz composite score for each diagnostic group (ASD—red, NT—sky-blue) across age-group (old, middle, young). Error bars denote standard error. Scatter plot showing the relationship between age and Schwarz composite score for ASD **(b)** and NT **(c)** in each age-group (blue: old, yellow—middle, gray—young). Centered-age (age—mean age in each age-group) was calculated for visualization only. Statistical models used raw age. Solid lines represent the linear regression fit for each group with shaded 95% confidence interval.

Figure 2a shows mean Schwarz composite values for each age-group. For visualization purposes, centered-age (Figures 2b,c) was calculated using the following Equation 2:


$$\mathrm{Ag}e_{\text{centered}}=\mathrm{Age}-{\bar{M}}_{\mathrm{age}-\text{group}}$$ (2)

where ${\bar{M}}_{\mathrm{age}-\text{group}}$ is the mean age in each age-group. No diagnostic difference emerged in any age-group [old: t(55.75) = 1.321, *p* = 0.186; middle: t(70.99) = 0.342, *p* = 0.726; young: t(76.95) = 0.331, *p* = 0.743]. Separate regression analyses were then run for each age group, using age and diagnostic group as independent variables. Age effects were not significant for the old ( β = 0.0002, *t* = 0.064, *p* = 0.961) or middle age-groups ( β = − 0.0003, *t* = −0.061, *p* = 0.902) but trended toward significance in the young age-group ( β = − 0.012, *t* = −1.84, *p* = 0.064). No effects of diagnostic group or diagnostic group x age interaction were found for any age-group (old: diagnostic group *p* = 0.186, interaction *p* = 0.286; middle: diagnostic group *p* = 0.571, interaction *p* = 0.573; young: diagnostic group *p* = 0.804, interaction *p* = 0.941). The regression analysis within each diagnostic group found similar age effects across ASD and NT groups except for the old age-group. Figures 2b,c show that in the old age-group, a significant negative relationship between age and the Schwartz composite was evident in the NT group ( β = − 0.004, *t* = −2.73, *p* = 0.007) but not in the ASD group ( β = − 0.001, *t* = −0.592, *p* = 0.566). In the middle age-group, neither the ASD nor the NT group showed age effects (ASD: β = − 0.001, *t* = −0.458, *p* = 0.595; NT: β = − 0.004, *t* = −1.37, *p* = 0.184). In the young age-group both ASD and NT groups showed significant age effects, with the negative slope revealing a reduction in cortical thickness with an increase in age (ASD: β = − 0.11, *t* = −2.70, *p* = 0.006; NT: β = − 0.011, *t* = −2.91, *p* = 0.008).

Given that dementia predominantly manifests later in adulthood, subsequent analyses focused on the old age-group. ROI-wise regression of the Schwarz composite on age (Supplementary Table 3) showed that, in the ASD group, the majority of regions did not demonstrate a significant age-related slope (*p*s > 0.10). Supplementary Table 3 shows statistics for each region and Supplementary Figure 1 shows regression plots for each group for each region. In the ASD group, the bilateral middle temporal gyrus approached significance (Supplementary Figure 1c, left: β = − 0.008, *t* = −2.45, *p* = 0.019; right: β = − 0.005, *t* = −1.97, *p* = 0.063) but did not survive the FDR correction (left *p*_FDR_ = 0.228; right *p*_FDR_ = 0.377). In the NT group, robust age effects were observed in the left middle temporal gyrus (Supplementary Figure 1c left, β = − 0.008, *t* = −3.24, *p* = 0.002, *p*_FDR_ = 0.029), right middle temporal gyrus (Supplementary Figure 1c right, β = − 0.007, *t* = −3.02, *p* = 0.005, *p*_FDR_ = 0.032), right inferior parietal cortex (Supplementary Figure 1d right, β = − 0.006, *t* = −2.25, *p* = 0.025, *p*_FDR_ = 0.091), and right fusiform gyrus (Supplementary Figure 1e right, β = − 0.005, *t* = −2.24, *p* = 0.030, *p*_FDR_ = 0.091).

To determine whether autism-specific traits may mask a potential age effect in the ASD group, the SRS-2 self-reported T-score and the RBS-R total score were considered as covariates in the regression models. In the ASD group, controlling for each measure did not alter the results for the Schwarz composite (SRS-2: β = − 0.001, *t* = −0.50, *p* = 0.615; RBS-R: β = − 0.001, *t* = −0.66, *p* = 0.571). In the NT group, the age-related decline remained significant after adjusting for SRS-2 ( β = − 0.003, *t* = −2.24, *p* = 0.031) and RBS-R score ( β = − 0.003, *t* = −2.64, *p* = 0.010). Models for individual ROIs revealed that, after adjusting for SRS score, the ASD group retained a significant age-related slope only in the bilateral middle temporal gyri (left: β = − 0.008, *t* = −2.41, *p* = 0.024, *p*_FDR_ = 0.168; right: β = − 0.006, *t* = −2.29, *p* = 0.028, *p*_FDR_ = 0.168). When RBS-R total score was added as a covariate, the bilateral middle temporal regions (left: β = − 0.008, *t* = −2.49, *p* = 0.008, *p*_FDR_ = 0.096; right: β = − 0.005, *t* = −1.98, *p* = 0.059, *p*_FDR_ = 0.354) remained significant in ASD group. To remain consistent in our approach for each group we also considered these covariates in the NT group. The age effect remained significant when SRS score was controlled for in the bilateral middle temporal gyri (left: β = − 0.007, *t* = −2.72, *p* = 0.013, *p*_FDR_ = 0.078; right: β = − 0.006, *t* = −2.67, *p* = 0.011, *p*_FDR_ = 0.078). After controlling for RBS-R, the age effect remained significant in left middle temporal gyrus ( β = − 0.008, *t* = −2.49, *p* = 0.008, *p*_FDR_ = 0.096), right middle temporal gyrus ( β = − 0.005, *t* = −1.98, *p* = 0.059, *p*_FDR_ = 0.354), right inferior parietal cortex ( β = − 0.006, *t* = −2.22, *p* = 0.032, *p*_FDR_ = 0.102), and right fusiform gyrus ( β = − 0.005, *t* = −2.21, *p* = 0.034, *p*_FDR_ = 0.102).

All analyses were repeated with FSIQ as an additional covariate. Inclusion of FSIQ did not modify any of the results (see Supplementary information and Supplementary Tables 4–7).

### 3.3 Brain-age

**Figure 3:**
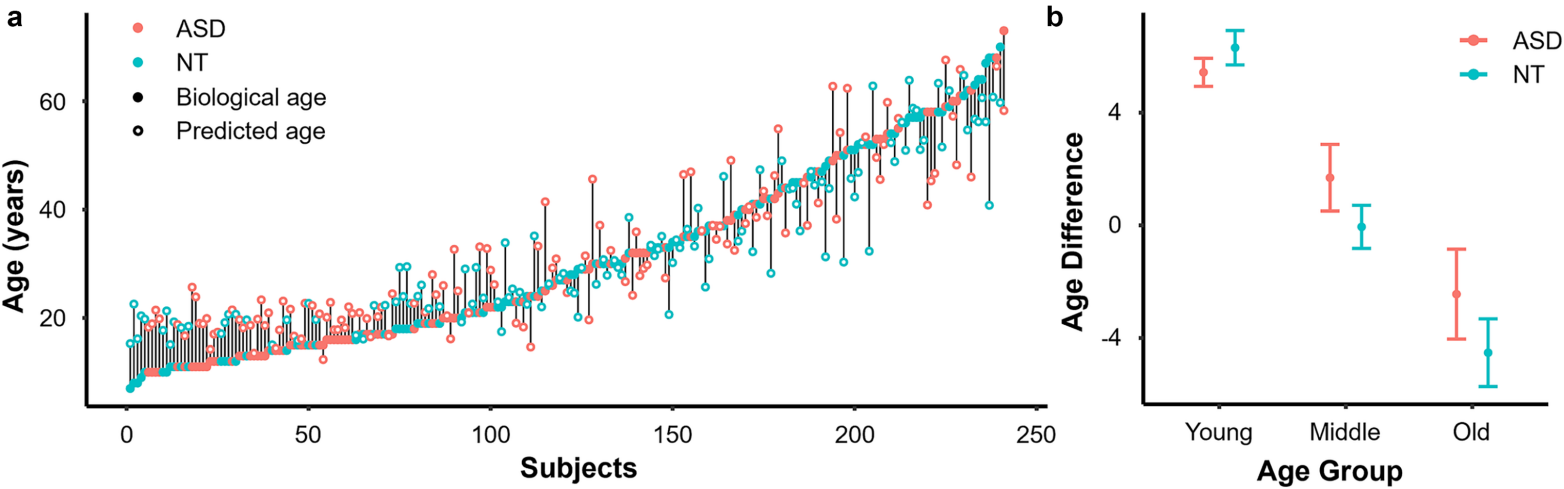
**(a)** Chronological and predicted age distribution across subjects (ASD—red, NT—sky blue, filled—chronological age, and empty—predicted age). **(b)** Mean age-difference for each diagnostic group (ASD—red, NT—sky-blue) across age-group (old, middle, young). Error bars denote standard error.

Figure 3a displays a distribution of chronological age and predicted age and Figure 3b shows mean ΔAge (predicted brain-age – chronological age) for each diagnostic group. Across the entire sample, the ΔAge did not differ significantly between ASD and NT groups, *F*(1, 238) = 1.65, *p* = 0.195. Sub-group analyses within each age-group found no diagnostic group effect, old: *F*(1, 67) = 0.850, *p* = 0.367; middle: *F*(1, 76) = 2.353, *p* = 0.122; young: *F*(1, 89) = 0.572, *p* = 0.447. Including FSIQ as a covariate did not modify these findings for either the whole cohort [*F*(1, 237) = 1.991, *p* = 0.159] nor within the different age-groups [old: *F*(1, 66) = 0.827, *p* = 0.373; middle: *F*(1, 75) = 2.09, *p* = 0.145; young: *F*(1, 88) = 0.079, *p* = 0.782].

## 4 Discussion

Previous epidemiological studies report an increased risk of early-onset dementia among autistic individuals (Klein et al., 2023; Vivanti et al., 2021). The current study investigated potential differences in an AD-sensitive structural brain composite across the lifespan in an ASD cohort relative to NT controls. The Schwarz composite and brain age were not significantly different between diagnostic groups when analyses considered the entire cohort, nor when the cohort was split into subgroups based on age. However, age-effects were found for both groups, with negative relationships revealing a decrease in cortical thickness with increasing age. Subgroup analyses showed that the same negative relationship was evident in the old NT group but was absent in the old ASD group, even when controlling for clinical factors. Secondary analyses controlling for FSIQ did not alter the overall pattern of findings. Our observations highlight the limitations of current AD-sensitive composites in assessing neurodegenerative risk in ASD, especially in older autistic adults.

Across the entire cohort, we did not observe significant differences between the ASD and NT groups in the Schwarz composite or in brain-age. Although the Schwarz composite declined with age across all participants, the overall group difference and the age trajectories were comparable between the two groups, consistent with previous studies (Bessé et al., 2025; Koolschijn and Geurts, 2016; Nunes et al., 2020). For example, Koolschijn and Geurts (2016) examined autistic and non-autistic adults aged 30 to 73 years old, and found comparable age-related effects in both groups. However, subgroup analyses in the present study revealed an age-related reduction in the Schwartz composite in the old NT group. Conversely, the old ASD group demonstrated a relative preservation of this metric across most regions vulnerable to AD pathology. Although cross sectional data cannot definitively characterize individual trajectories, our finding parallels recent diffusion MRI results indicating that while NT exhibit age-related reductions in white matter microstructure, these age-associated differences are not present in autistic adults (Shin et al., 2025). The weaker association between age and the Schwartz composite in the old ASD group can be interpreted in several ways. First, preserved structure later in life may reflect unique, potentially “safeguarded” neurodevelopmental trajectories in ASD, resulting in an attenuated rate of change in older adulthood (Mensen et al., 2017; van Rooij et al., 2018; Bessé et al., 2025). However, the increased prevalence of dementia in autistic adults runs counter to this argument (Croen et al., 2015; Hand et al., 2020; Vivanti et al., 2021). Second, the neurodegenerative pattern associated with aging and dementia risk in ASD may not follow the same neurodegenerative pattern as in NT’s. Third, lifelong atypical development may be associated with cortical thinning earlier in life and then plateaued. However, cortical thickness measures in the ASD group did not significantly deviate from the range of the NT group (95% confidence interval of Schwarz composite in ASD = [2.63 2.70] and NT = [2.66 2.71]) providing evidence against an early-plateau interpretation. Fourth, the inclusion of autistic adults without intellectual impairment introduces the possibility of sampling bias or survivor bias. Nevertheless, the substantial variability in the ASD data argues against a universal protective effect by cognitive reserve.

Our findings align with studies supporting the parallel or safeguard development theory in ASD suggesting that autistic adults may reflect relative resilience to age-related decline or exhibit patterns parallel to neurotypical adults (Mensen et al., 2017; van Rooij et al., 2018; Bessé et al., 2025) and highlight the complexity of structural brain differences in this population. This contrasts prior evidence of divergent brain structure trajectories between ASD and NT (Khundrakpam et al., 2017; Zielinski et al., 2014). However, our study differs from previous studies in three important ways. First, our cohort included individuals over the age of 50, providing insight into structural brain alterations in older autistic individuals. Second, we calculated biological brain-age, suggesting that atypical structure in ASD may not necessarily reflect conventional accelerated aging in the brain. Finally, we employed whole-brain analyses for brain-age, rather than focusing on specific cortical or subcortical regions. Nevertheless, previous studies have established that various neurodegenerative and genetic conditions, such as dementia, mild cognitive impairment (MCI), and Down syndrome, exhibit accelerated brain age, indicating an advanced pattern of structural brain aging (Biondo et al., 2022; Cole et al., 2017; Franke and Gaser, 2019; Seitz-Holland et al., 2024). In contrast, our findings suggest that brain aging in ASD is not globally accelerated. Notably in the old cohort, the majority of Schwarz composite regions demonstrated no significant age-related decline in the ASD group, whereas the NT group showed clear age-related reductions. This suggests that brain structure in ASD is not characterized by a global pattern of age-related atrophy, but is instead associated with distinct regions that have been shown to differ structurally from those in neurotypical individuals. Therefore, the traditional measures of brain age, which are effective in capturing diffuse brain atrophy in neurodegenerative and genetic conditions, may not be as sensitive to the unique regional, and potentially “protected” patterns observed in the ASD population.

Our observations highlight the limitations of current dementia-sensitive composites in assessing neurodegenerative risk in ASD. The Schwarz composite was developed using data from older neurotypical adults and individuals with AD (Schwarz et al., 2016). Refining existing dementia-sensitive measures for populations with neurodevelopmental disorders, particularly for autistic individuals, should be a focus for future studies. The heterogeneity of ASD, encompassing genetic, epigenetic, and phenotypic variability, further complicates the identification of consistent neuroanatomical biomarkers. Additionally, the developmental trajectories of brain regions in ASD differ from those of neurotypical individuals, potentially obscuring patterns indicative of dementia risk. Elevated levels of soluble amyloid precursor protein *α* (sAPP-α) have been reported in individuals with autism (Bailey et al., 2012; Ray et al., 2011). Given that sAPP-α exerts a potent neuroprotective effect by preventing amyloid-*β* -induced loss of dendritic spines and reducing tau hyperphosphorylation (Furukawa et al., 1996; Tackenberg and Nitsch, 2019), its upregulation may alter the trajectory of neurodegeneration. Consequently, dementia-related structural brain alternations in autism population may diverge from those observed in neurotypical cohorts. Lastly the Schwarz composite is specifically calibrated to detect atrophy associated with AD (Schwarz et al., 2016). Given the elevated risk of dementia diagnosis in the autistic population (Croen et al., 2015; Hand et al., 2020; Vivanti et al., 2021), the absence of an age-related decline in these AD-sensitive regions suggests that if autistic adults are indeed more vulnerable to AD dementia, the brain markers developed in NT cohorts may not capture this vulnerability. Moreover, it is possible that the increased dementia prevalence in ASD could be driven by non-AD pathologies, such as frontotemporal or vascular dementia, which are not captured by the AD-specific marker implemented in the current study. Dementia sub-typing in ASD is an important avenue for future studies.

The current study benefits from a large sample size that includes adolescents, young adults and middle to older-aged adults allowing for the examination of dementia sensitive composites in a diverse autistic population. However, several limitations should be considered. First, individuals in the current study did not complete formal clinical evaluation for dementia using standardized cognitive assessments (e.g., Clinical Dementia Rating or Montreal Cognitive Assessment), preventing insight into whether individuals with thinner cortex were closer to clinical impairment. Second, our cohort included autistic individuals without intellectual impairment (FSIQ > 100, see Table 1). Considering the higher prevalence of early-onset dementia in autistic individuals with intellectual disability compared to those without intellectual disability (Vivanti et al., 2021), our cohort may have been biased against autistic individuals with MCI or AD, which could limit the generalizability of findings to the broader ASD population. However, it is important to note that our NT group was neurologically healthy and still showed age-related neurodegeneration in these AD-dementia regions; suggesting that dementia related diagnosis is not necessary to identify age related cortical thinning. Third, the wide age range of participants (7 to 73 years old) may have introduced variability, as dementia-related brain atrophy may not emerge until later in life. Fourth, our findings did not reveal significant interactions between age and group, but this should be interpreted cautiously given the cross-sectional study design. The inability to track neurodevelopmental trajectories means that high inter-individual variability and cohort effects could mask the late-life emergence of divergent atrophy patterns. Fifth, although the use of non-parametric permutation tests provides confidence in our null finding, the relatively small sample size and insufficient statistical power may have also contributed to this.

Future research on dementia in ASD should prioritize developing dementia-sensitive composites that are specifically tailored for the ASD population. For example, utilizing standardized clinical assessments to evaluate dementia status alongside cohorts that include a more representative range of cognitive abilities may better capture the potential overlap between ASD and dementia-related neurodegeneration. Additionally, longitudinal studies are particularly important for capturing intra-individual changes and providing insights into age-related trajectories in ASD. Finally, multimodal approaches that are sensitive to different dementia subtypes and combine neuroimaging, genetic, biofluid and clinical data may offer a more comprehensive understanding of ASD and enable the identification of clinically significant biomarkers to support personalized interventions (DeSimone et al., 2024; Wang et al., 2023).

## 5 Conclusion

Established structural brain composites that are sensitive to age and AD-dementia risk in NT cohorts may not be helpful in understanding aging and dementia risk in older autistic adults without intellectual impairment. By addressing the inherent heterogeneity of ASD and employing comprehensive biomarkers sensitive to a broader spectrum of neurodegenerative pathologies, future research can better elucidate the relationship between autism and dementia, ultimately advancing clinical care for this population.

## Data Availability

The original contributions presented in the study are included in the article/Supplementary material, further inquiries can be directed to the corresponding author.

## Footnotes

1
https://www.sfari.org/resource/research-match/

2
http://surfer.nmr.mgh.harvard.edu

3
https://github.com/Jfortin1/ComBatHarmonization

4
https://github.com/james-cole/brainageR

5
https://www.fil.ion.ucl.ac.uk/spm/software/spm12

6
https://CRAN.R-project.org/package=kernlab
