# Supplementary material for: Neuroanatomical patterns of dementia risk in autism spectrum disorder
Source: Front Aging Neurosci. 2026 Apr 2;18:1771822. doi: 10.3389/fnagi.2026.1771822 (PMC13082925; doi:10.3389/fnagi.2026.1771822)

*Supplementary Material*

# Supplementary Tables

**Supplementary Table 1.** Demographic information of each site

| University of Florida |  |  |  |  |
| --- | --- | --- | --- | --- |
|  | ASD  Mean (SD) | NT Mean (SD) | t/**χ^2^** | p |
| N | 45 | 60 |  |  |
| Age (years)  Range | 46.18 (11.03) 30 – 73 | 47.53 (11.68) 30 – 70 | 0.608 | 0.545 |
| Sex (M/F)^a^ | 27 / 18 | 28 / 32 | 1.337 | 0.248 |
| % of female | 40.0 % | 53.3 % |  |  |
| Full-scale IQ | 107.3 (13.67) | 108.0 (11.85) | 0.242 | 0.809 |
| Verbal IQ | 107.7 (13.52) | 106.2 (11.00) | 0.606 | 0.546 |
| Performance IQ | 105.0 (13.89) | 108.0 (14.09) | 1.078 | 0.284 |
| ADOS-2 (total raw)^b^ | 10.81 (3.293) | - | - | - |
| Total intracranial volume (cm^3^) | 1536 (166.0) | 1502 (161.4) | 1.047 | 0.298 |
|  |  |  |  |  |
| University of Kansas |  |  |  |  |
|  | ASD  Mean (SD) | NT Mean (SD) | t/**χ^2^** | p |
| N | 58 | 40 |  |  |
| Age (years)  Range | 16.48 (5.63) 10 – 33 | 17.52 (7.28) 7 – 34 | 0.762 | 0.448 |
| Sex (M/F)^a^ | 38 / 20 | 17 / 23 | 4.201 | **0.040** |
| % of female | 34.5 % | 57.5 % |  |  |
| Full-scale IQ | 103.6 (16.06) | 111.6 (13.31) | 2.666 | **0.009** |
| Verbal IQ^c^ | 100.7 (16.20) | 109.0 (11.39) | 0.548 | 0.585 |
| Performance IQ^c^ | 104.0 (16.08) | 110.0 (12.49) | 0.358 | 0.721 |
| ADOS-2 (CSS)^d^ | 6.067 (2.434) | - | - | - |
| Total intracranial volume (cm^3^) | 1571 (160.9) | 1525 (155.1) | 1.426 | 0.157 |
|  |  |  |  |  |
| University of Southwestern |  |  |  |  |
|  | ASD  Mean (SD) | NT Mean (SD) | t/**χ^2^** | p |
| N | 19 | 19 |  |  |
| Age (years)  Range | 22.37 (8.35) 15 – 49 | 21.74 (5.83) 11 – 33 | 0.270 | 0.789 |
| Sex (M/F)^a^ | 18 / 1 | 17 / 2 | 0 | 1 |
| % of female | 5.3 % | 10.5 % |  |  |
| Full-scale IQ | 106.3 (17.63) | 116.8 (12.61) | 2.117 | **0.042** |
| Verbal IQ | 103.6 (21.30) | 115.6 (12.62) | 2.113 | **0.043** |
| Performance IQ | 108.2 (14.11) | 113.9 (12.55) | 1.312 | 0.198 |
| ADOS-2 (CSS)^f^ | 6.333 (2.887) | - | - | - |
| Total intracranial volume (cm^3^) | 1666 (146.3) | 1584 (165.3) | 1.622 | 0.114 |

a Chi-square (χ^2^) statistics

b ADOS-2 total raw score was used for those ages over 30 including 42 participants

c included 44 in ASD group and 30 in NT group

d ADOS-2 CSS score was used for whose age less than 31 including 30 participants

f ADOS-2 CSS score was used for whose age less than 31 including 3 participants

**Supplementary Table 2.** Age associations with Schwarz composite ROIs

|  | ASD | | | |  | NT | | | |
| --- | --- | --- | --- | --- | --- | --- | --- | --- | --- |
|  | $\beta$ | t | *p* | *p*_FDR_ |  | $\beta$ | t | *p* | *p*_FDR_ |
| Left entorhinal cortex | 0.002 | 1.43 | 0.150 | 0.159 |  | 0.004 | 2.97 | **0.001** | **0.001** |
| Right entorhinal cortex | 0.003 | 1.44 | 0.159 | 0.159 |  | 0.004 | 3.39 | **0.001** | **0.001** |
| Left inferior temporal gyrus | -0.005 | -6.72 | **< 0.001** | **< 0.001** |  | -0.004 | -5.60 | **< 0.001** | **< 0.001** |
| Right inferior temporal gyrus | -0.004 | -6.12 | **< 0.001** | **< 0.001** |  | -0.003 | -4.98 | **< 0.001** | **< 0.001** |
| Left middle temporal gyrus | -0.008 | -10.57 | **< 0.001** | **< 0.001** |  | -0.007 | -11.48 | **< 0.001** | **< 0.001** |
| Right middle temporal gyrus | -0.007 | -9.52 | **< 0.001** | **< 0.001** |  | -0.006 | -10.49 | **< 0.001** | **< 0.001** |
| Left inferior parietal cortex | -0.006 | -9.22 | **< 0.001** | **< 0.001** |  | -0.005 | -8.04 | **< 0.001** | **< 0.001** |
| Right inferior parietal cortex | -0.006 | -9.11 | **< 0.001** | **< 0.001** |  | -0.006 | -8.66 | **< 0.001** | **< 0.001** |
| Left fusiform gyrus | -0.004 | -6.52 | **< 0.001** | **< 0.001** |  | -0.002 | -4.09 | **< 0.001** | **< 0.001** |
| Right fusiform gyrus | -0.004 | -5.70 | **< 0.001** | **< 0.001** |  | -0.003 | -4.54 | **< 0.001** | **< 0.001** |
| Left precuneus | -0.006 | -8.71 | **< 0.001** | **< 0.001** |  | -0.006 | -9.44 | **< 0.001** | **< 0.001** |
| Right precuneus | -0.006 | -9.51 | **< 0.001** | **< 0.001** |  | -0.005 | -8.15 | **< 0.001** | **< 0.001** |

**Supplementary Table 3.** Age associations with Schwarz composite ROIs in the old age-group

|  | ASD | | | |  | NT | | | |
| --- | --- | --- | --- | --- | --- | --- | --- | --- | --- |
|  | $\beta$ | t | *p* | *p*_FDR_ |  | $\beta$ | t | *p* | *p*_FDR_ |
| Left entorhinal cortex | 0.006 | 0.94 | 0.376 | 0.803 |  | 0.002 | 0.39 | 0.706 | 0.706 |
| Right entorhinal cortex | 0.003 | 0.61 | 0.531 | 0.803 |  | -0.006 | -1.39 | 0.164 | 0.236 |
| Left inferior temporal gyrus | -0.001 | -0.50 | 0.610 | 0.803 |  | -0.002 | -0.77 | 0.466 | 0.551 |
| Right inferior temporal gyrus | -0.002 | -0.73 | 0.505 | 0.803 |  | -0.001 | -0.60 | 0.675 | 0.706 |
| Left middle temporal gyrus | -0.008 | -2.45 | **0.019** | 0.228 |  | -0.008 | -3.20 | **0.003** | **0.026** |
| Right middle temporal gyrus | -0.005 | -1.97 | 0.063 | 0.377 |  | -0.007 | -3.09 | **0.004** | **0.026** |
| Left inferior parietal cortex | 0.000 | -0.08 | 0.902 | 0.902 |  | -0.004 | -1.78 | 0.081 | 0.131 |
| Right inferior parietal cortex | -0.003 | -1.68 | 0.106 | 0.422 |  | -0.006 | -2.31 | **0.021** | 0.069 |
| Left fusiform gyrus | -0.001 | -0.58 | 0.562 | 0.803 |  | -0.002 | -1.32 | 0.194 | 0.253 |
| Right fusiform gyrus | -0.001 | -0.49 | 0.632 | 0.803 |  | -0.005 | -2.29 | **0.027** | 0.070 |
| Left precuneus | 0.000 | -0.22 | 0.843 | 0.902 |  | -0.005 | -2.02 | **0.047** | 0.102 |
| Right precuneus | -0.001 | -0.42 | 0.669 | 0.803 |  | -0.004 | -1.79 | 0.070 | 0.130 |

**Supplementary Table 4.** Age associations with Schwarz composite ROIs with controlling FSIQ

|  | ASD | | | |  | NT | | | |
| --- | --- | --- | --- | --- | --- | --- | --- | --- | --- |
|  | $\beta$ | t | *p* | *p*_FDR_ |  | $\beta$ | t | *p* | *p*_FDR_ |
| Left entorhinal cortex | 0.002 | 1.17 | 0.228 | 0.249 |  | 0.004 | 3.20 | **0.001** | **0.001** |
| Right entorhinal cortex | 0.002 | 1.10 | 0.287 | 0.287 |  | 0.004 | 3.36 | **0.002** | **0.002** |
| Left inferior temporal gyrus | -0.005 | -6.82 | **< 0.001** | **< 0.001** |  | -0.003 | -5.25 | **< 0.001** | **< 0.001** |
| Right inferior temporal gyrus | -0.004 | -6.23 | **< 0.001** | **< 0.001** |  | -0.003 | -4.92 | **< 0.001** | **< 0.001** |
| Left middle temporal gyrus | -0.008 | -10.76 | **< 0.001** | **< 0.001** |  | -0.006 | -11.16 | **< 0.001** | **< 0.001** |
| Right middle temporal gyrus | -0.007 | -9.57 | **< 0.001** | **< 0.001** |  | -0.006 | -10.30 | **< 0.001** | **< 0.001** |
| Left inferior parietal cortex | -0.006 | -9.12 | **< 0.001** | **< 0.001** |  | -0.005 | -7.69 | **< 0.001** | **< 0.001** |
| Right inferior parietal cortex | -0.006 | -9.08 | **< 0.001** | **< 0.001** |  | -0.006 | -8.44 | **< 0.001** | **< 0.001** |
| Left fusiform gyrus | -0.004 | -6.68 | **< 0.001** | **< 0.001** |  | -0.002 | -3.75 | **< 0.001** | **< 0.001** |
| Right fusiform gyrus | -0.004 | -5.87 | **< 0.001** | **< 0.001** |  | -0.003 | -4.15 | **< 0.001** | **< 0.001** |
| Left precuneus | -0.006 | -8.87 | **< 0.001** | **< 0.001** |  | -0.006 | -9.10 | **< 0.001** | **< 0.001** |
| Right precuneus | -0.006 | -9.37 | **< 0.001** | **< 0.001** |  | -0.005 | -7.79 | **< 0.001** | **< 0.001** |

**Supplementary Table 5.** Age associations with Schwarz composite ROIs in the old age-group with controlling FSIQ

|  | ASD | | | |  | NT | | | |
| --- | --- | --- | --- | --- | --- | --- | --- | --- | --- |
|  | $\beta$ | t | *p* | *p*_FDR_ |  | $\beta$ | t | *p* | *p*_FDR_ |
| Left entorhinal cortex | 0.005 | 0.80 | 0.411 | 0.831 |  | 0.001 | 0.29 | 0.777 | 0.777 |
| Right entorhinal cortex | 0.002 | 0.40 | 0.705 | 0.833 |  | -0.006 | -1.36 | 0.172 | 0.247 |
| Left inferior temporal gyrus | -0.002 | -0.48 | 0.625 | 0.831 |  | -0.002 | -0.74 | 0.439 | 0.519 |
| Right inferior temporal gyrus | -0.001 | -0.47 | 0.623 | 0.831 |  | -0.001 | -0.54 | 0.612 | 0.663 |
| Left middle temporal gyrus | -0.007 | -2.15 | **0.039** | 0.426 |  | -0.008 | -3.24 | **0.002** | **0.031** |
| Right middle temporal gyrus | -0.005 | -1.88 | 0.066 | 0.426 |  | -0.007 | -3.02 | **0.005** | **0.035** |
| Left inferior parietal cortex | 0.001 | 0.26 | 0.843 | 0.843 |  | -0.004 | -1.74 | 0.083 | 0.143 |
| Right inferior parietal cortex | -0.003 | -1.51 | 0.134 | 0.582 |  | -0.006 | -2.25 | **0.025** | 0.079 |
| Left fusiform gyrus | -0.001 | -0.58 | 0.574 | 0.831 |  | -0.002 | -1.34 | 0.190 | 0.247 |
| Right fusiform gyrus | -0.001 | -0.51 | 0.624 | 0.831 |  | -0.005 | -2.24 | **0.030** | 0.079 |
| Left precuneus | -0.001 | -0.22 | 0.829 | 0.843 |  | -0.004 | -1.94 | 0.065 | 0.141 |
| Right precuneus | -0.001 | -0.43 | 0.639 | 0.831 |  | -0.004 | -1.72 | 0.088 | 0.143 |

**Supplementary Table 6.** Age associations with Schwarz composite ROIs in the old age-group with controlling SRS self T-score and FSIQ

|  | ASD | | | |  | NT | | | |
| --- | --- | --- | --- | --- | --- | --- | --- | --- | --- |
|  | $\beta$ | t | *p* | *p*_FDR_ |  | $\beta$ | t | *p* | *p*_FDR_ |
| Left entorhinal cortex | 0.006 | 1.011 | 0.309 | 0.731 |  | 0.001 | 0.137 | 0.922 | 0.922 |
| Right entorhinal cortex | 0.006 | 0.968 | 0.353 | 0.731 |  | -0.005 | -0.975 | 0.321 | 0.465 |
| Left inferior temporal gyrus | -0.002 | -0.581 | 0.584 | 0.731 |  | -0.002 | -0.724 | 0.452 | 0.542 |
| Right inferior temporal gyrus | -0.001 | -0.364 | 0.687 | 0.731 |  | -0.002 | -0.635 | 0.543 | 0.592 |
| Left middle temporal gyrus | -0.008 | -2.411 | **0.024** | 0.168 |  | -0.007 | -2.723 | **0.013** | 0.078 |
| Right middle temporal gyrus | -0.006 | -2.294 | **0.028** | 0.168 |  | -0.006 | -2.665 | **0.011** | 0.078 |
| Left inferior parietal cortex | -0.001 | -0.320 | 0.728 | 0.731 |  | -0.002 | -1.049 | 0.313 | 0.465 |
| Right inferior parietal cortex | -0.004 | -1.766 | 0.093 | 0.372 |  | -0.005 | -1.663 | 0.101 | 0.296 |
| Left fusiform gyrus | -0.001 | -0.508 | 0.596 | 0.731 |  | -0.002 | -0.962 | 0.349 | 0.465 |
| Right fusiform gyrus | -0.001 | -0.335 | 0.731 | 0.731 |  | -0.004 | -1.806 | 0.079 | 0.296 |
| Left precuneus | -0.001 | -0.470 | 0.657 | 0.731 |  | -0.004 | -1.488 | 0.138 | 0.296 |
| Right precuneus | -0.001 | -0.401 | 0.693 | 0.731 |  | -0.004 | -1.437 | 0.148 | 0.296 |

**Supplementary Table 7.** Age associations with Schwarz composite ROIs in the old age-group with controlling RBS-R score and FSIQ

|  | ASD | | | |  | NT | | | |
| --- | --- | --- | --- | --- | --- | --- | --- | --- | --- |
|  | $\beta$ | t | *p* | *p*_FDR_ |  | $\beta$ | t | *p* | *p*_FDR_ |
| Left entorhinal cortex | 0.006 | 0.883 | 0.378 | 0.680 |  | 0.002 | 0.442 | 0.704 | 0.704 |
| Right entorhinal cortex | 0.004 | 0.599 | 0.563 | 0.680 |  | -0.006 | -1.384 | 0.171 | 0.257 |
| Left inferior temporal gyrus | -0.002 | -0.537 | 0.591 | 0.680 |  | -0.002 | -0.731 | 0.437 | 0.524 |
| Right inferior temporal gyrus | -0.002 | -0.649 | 0.508 | 0.680 |  | -0.001 | -0.504 | 0.644 | 0.703 |
| Left middle temporal gyrus | -0.008 | -2.486 | **0.008** | 0.096 |  | -0.007 | -3.107 | **0.002** | **0.024** |
| Right middle temporal gyrus | -0.005 | -1.981 | 0.059 | 0.354 |  | -0.007 | -3.002 | **0.004** | **0.024** |
| Left inferior parietal cortex | -0.001 | -0.357 | 0.761 | 0.761 |  | -0.004 | -1.686 | 0.096 | 0.165 |
| Right inferior parietal cortex | -0.003 | -1.605 | 0.119 | 0.476 |  | -0.006 | -2.217 | **0.032** | 0.102 |
| Left fusiform gyrus | -0.002 | -0.616 | 0.564 | 0.680 |  | -0.002 | -1.294 | 0.198 | 0.264 |
| Right fusiform gyrus | -0.001 | -0.502 | 0.609 | 0.680 |  | -0.005 | -2.210 | **0.034** | 0.102 |
| Left precuneus | -0.001 | -0.478 | 0.623 | 0.680 |  | -0.004 | -1.923 | 0.062 | 0.149 |
| Right precuneus | -0.002 | -0.649 | 0.535 | 0.680 |  | -0.004 | -1.686 | 0.090 | 0.165 |

# Statistical analyses in Schwarz composite with FSIQ

1. ANCOVA with group (ASD vs NT) as independent variable and FSIQ as covariate

- Group: F(1,238) = 1.98, *p* = 0.161

1. Regression analysis with age and group as independent variables and FSIQ as covariate

- Group: $\beta$ = -0.021, t = -1.66, *p* = 0.091
- Age: $\beta$ = -0.005, t = -6.15, *p* < 0.001
- Group x Age interaction: $\beta$= -0.0006, t = 1.81, *p* = 0.075

1. Regression analysis with age as independent variable and FSIQ as covariate within ASD group

- Age: $\beta$ = -0.004, t = -7.92, *p* < 0.001

1. Regression analysis with age as independent variable and FSIQ as covariate within NT group

- Age: $\beta$ = -0.003, t = -6.71, *p* < 0.001

# Supplementary Figure


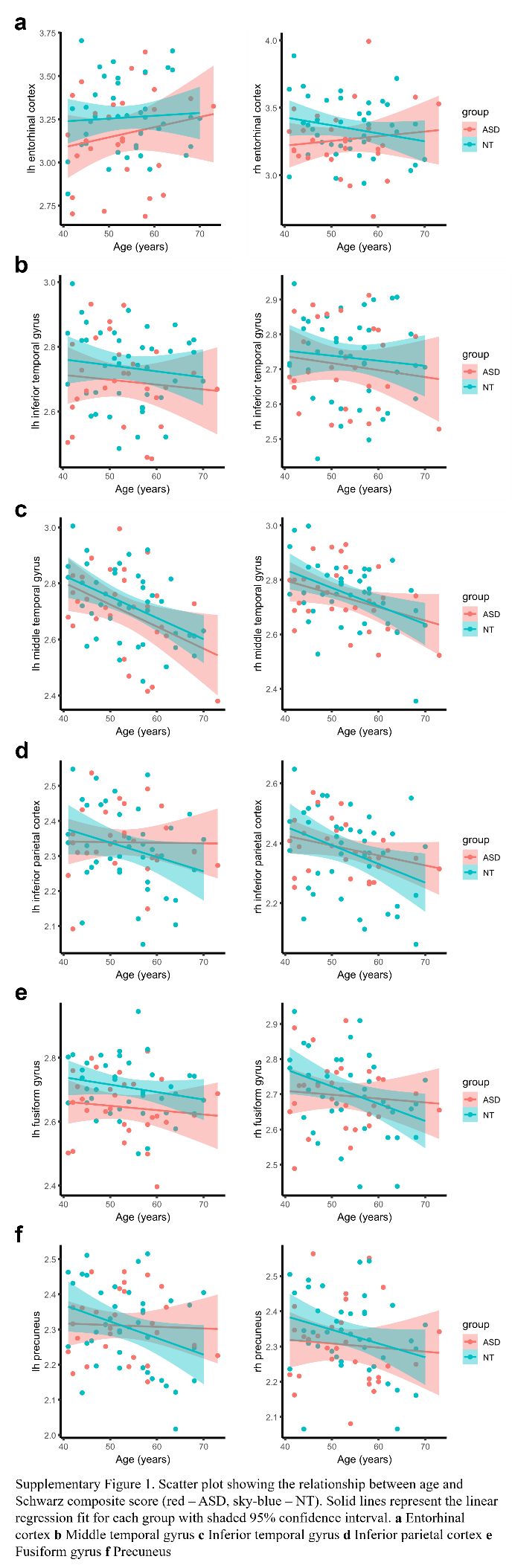

Supplement: Supplementary file 1 [file Data_Sheet_1.docx]
